# Predictors for extubation failure in COVID-19 patients using a machine learning approach

**DOI:** 10.1186/s13054-021-03864-3

**Published:** 2021-12-27

**Authors:** Lucas M. Fleuren, Tariq A. Dam, Michele Tonutti, Daan P. de Bruin, Robbert C. A. Lalisang, Diederik Gommers, Olaf L. Cremer, Rob J. Bosman, Sander Rigter, Evert-Jan Wils, Tim Frenzel, Dave A. Dongelmans, Remko de Jong, Marco Peters, Marlijn J. A. Kamps, Dharmanand Ramnarain, Ralph Nowitzky, Fleur G. C. A. Nooteboom, Wouter de Ruijter, Louise C. Urlings-Strop, Ellen G. M. Smit, D. Jannet Mehagnoul-Schipper, Tom Dormans, Cornelis P. C. de Jager, Stefaan H. A. Hendriks, Sefanja Achterberg, Evelien Oostdijk, Auke C. Reidinga, Barbara Festen-Spanjer, Gert B. Brunnekreef, Alexander D. Cornet, Walter van den Tempel, Age D. Boelens, Peter Koetsier, Judith Lens, Harald J. Faber, A. Karakus, Robert Entjes, Paul de Jong, Thijs C. D. Rettig, Sesmu Arbous, Sebastiaan J. J. Vonk, Mattia Fornasa, Tomas Machado, Taco Houwert, Hidde Hovenkamp, Roberto Noorduijn Londono, Davide Quintarelli, Martijn G. Scholtemeijer, Aletta A. de Beer, Giovanni Cinà, Adam Kantorik, Tom de Ruijter, Willem E. Herter, Martijn Beudel, Armand R. J. Girbes, Mark Hoogendoorn, Patrick J. Thoral, Paul W. G. Elbers, the Dutch ICU Data Sharing Against Covid-19 Collaborators, Julia Koeter, Roger van Rietschote, M. C. Reuland, Laura van Manen, Leon Montenij, Jasper van Bommel, Roy van den Berg, Ellen van Geest, Anisa Hana, B. van den Bogaard, Peter Pickkers, Pim van der Heiden, Claudia van Gemeren, Arend Jan Meinders, Martha de Bruin, Emma Rademaker, Frits H. M. van Osch, Martijn de Kruif, Nicolas Schroten, Klaas Sierk Arnold, J. W. Fijen, Jacomar J. M. van Koesveld, Koen S. Simons, Joost Labout, Bart van de Gaauw, Michael Kuiper, Albertus Beishuizen, Dennis Geutjes, Johan Lutisan, Bart P. Grady, Remko van den Akker, Tom A. Rijpstra, W. G. Boersma, Daniel Pretorius, Menno Beukema, Bram Simons, A. A. Rijkeboer, Marcel Aries, Niels C. Gritters van den Oever, Martijn van Tellingen, Annemieke Dijkstra, Rutger van Raalte

**Affiliations:** 1 Department of Intensive Care Medicine, Laboratory for Critical Care Computational Intelligence, Amsterdam Medical Data Science, Amsterdam UMC, Vrije Universiteit, Amsterdam, The Netherlands; 2 Pacmed, Amsterdam, The Netherlands; 3 Department of Intensive Care, Erasmus Medical Center, Rotterdam, The Netherlands; 4 Department of Intensive Care, UMC Utrecht, Utrecht, The Netherlands; 5 ICU, OLVG, Amsterdam, The Netherlands; 6 Department of Anesthesiology and Intensive Care, St. Antonius Hospital, Nieuwegein, The Netherlands; 7 Department of Intensive Care, Franciscus Gasthuis and Vlietland, Rotterdam, The Netherlands; 8 Department of Intensive Care Medicine, Radboud University Medical Center, Nijmegen, The Netherlands; 9 Department of Intensive Care Medicine, Amsterdam UMC, Amsterdam, The Netherlands; 10 Intensive Care, Bovenij Ziekenhuis, Amsterdam, The Netherlands; 11 Intensive Care, Canisius Wilhelmina Ziekenhuis, Nijmegen, The Netherlands; 12 Intensive Care, Catharina Ziekenhuis Eindhoven, Eindhoven, The Netherlands; 13 Department of Intensive Care, ETZ Tilburg, Tilburg, The Netherlands; 14 Intensive Care, HagaZiekenhuis, Den Haag, The Netherlands; 15 Intensive Care, Laurentius Ziekenhuis, Roermond, The Netherlands; 16 Department of Intensive Care Medicine, Northwest Clinics, Alkmaar, The Netherlands; 17 Intensive Care, Reinier de Graaf Gasthuis, Delft, The Netherlands; 18 Intensive Care, Spaarne Gasthuis, Haarlem en Hoofddorp, The Netherlands; 19 Intensive Care, VieCuri Medisch Centrum, Venlo, The Netherlands; 20 Intensive Care, Zuyderland MC, Heerlen, The Netherlands; 21 Department of Intensive Care, Jeroen Bosch Ziekenhuis, Den Bosch, The Netherlands; 22 Intensive Care, Albert Schweitzerziekenhuis, Dordrecht, The Netherlands; 23 ICU, Haaglanden Medisch Centrum, Den Haag, The Netherlands; 24 ICU, Maasstad Ziekenhuis Rotterdam, Rotterdam, The Netherlands; 25 ICU, SEH, BWC, Martiniziekenhuis, Groningen, The Netherlands; 26 Intensive Care, Ziekenhuis Gelderse Vallei, Ede, The Netherlands; 27 Department of Intensive Care, Ziekenhuisgroep Twente, Almelo, The Netherlands; 28 Department of Intensive Care, Medisch Spectrum Twente, Enschede, The Netherlands; 29 Department of Intensive Care, Ikazia Ziekenhuis Rotterdam, Rotterdam, The Netherlands; 30 Antonius Ziekenhuis Sneek, Sneek, The Netherlands; 31 Intensive Care, Medisch Centrum Leeuwarden, Leeuwarden, The Netherlands; 32 ICU, IJsselland Ziekenhuis, Capelle Aan Den IJssel, The Netherlands; 33 ICU, WZA, Assen, The Netherlands; 34 Department of Intensive Care, Diakonessenhuis Hospital, Utrecht, The Netherlands; 35 Department of Intensive Care, Adrz, Goes, The Netherlands; 36 Department of Anesthesia and Intensive Care, Slingeland Ziekenhuis, Doetinchem, The Netherlands; 37 Department of Anesthesiology, Intensive Care and Pain Medicine, Amphia Ziekenhuis, Breda, The Netherlands; 38 Department of Intensive Care, LUMC, Leiden, The Netherlands; 39 BigData Republic, Nieuwegein, The Netherlands; 40 Department of Neurology, Amsterdam UMC, Universiteit Van Amsterdam, Amsterdam, The Netherlands; 41 Quantitative Data Analytics Group, Department of Computer Science, Faculty of Science, Vrije Universiteit, Amsterdam, The Netherlands; 42 Business Intelligence, Haaglanden MC, Den Haag, The Netherlands; 43 Department of Intensive Care Medicine, Amsterdam UMC, Universiteit Van Amsterdam, Amsterdam, The Netherlands; 44 Department of Intensive Care, BovenIJ Ziekenhuis, Amsterdam, The Netherlands; 45 Department of Anesthesiology, Pain Management and Intensive Care, Catharina Ziekenhuis Eindhoven, Eindhoven, The Netherlands; 46 Department of ICMT, Haga Ziekenhuis, Den Haag, The Netherlands; 47 Department of Intensive Care Medicine, Radboud University Medical Centre, Nijmegen, The Netherlands; 48 Department of Internal Medicine and Intensive Care, St Antonius Hospital, Nieuwegein, The Netherlands; 49 Department of Clinical Epidemiology, VieCuri Medisch Centrum, Venlo, The Netherlands; 50 Department of Pulmonology, Zuyderland MC, Heerlen, The Netherlands; 51 Department of Intensive Care, Diakonessenhuis Hospital, Utrecht, The Netherlands; 52 ICU, Maasstad Ziekenhuis, Rotterdam, The Netherlands; 53 Martiniziekenhuis, Groningen, The Netherlands; 54 Department of Information Technology, Slingeland Ziekenhuis, Doetinchem, The Netherlands; 55 Intensive Care, Adrz, Goes, The Netherlands; 56 Department of Pulmonology, Northwest Clinics, Alkmaar, The Netherlands; 57 Department of Intensive Care Medicine, Hospital St Jansdal, Harderwijk, The Netherlands; 58 Department of Intensive Care, Streekziekenhuis Koningin Beatrix, Winterswijk, The Netherlands; 59 Intensive Care, Bravis Ziekenhuis, Bergen Op Zoom en Roosendaal, The Netherlands; 60 ICU, Flevoziekenhuis, Almere, The Netherlands; 61 MUMC+, University Maastricht, Maastricht, The Netherlands; 62 Intensive Care, Treant Zorggroep, Emmen, The Netherlands; 63 Department of Intensive Care Medicine, Afdeling Intensive Care, Ziekenhuis Tjongerschans, Heerenveen, The Netherlands; 64 Department of Intensive Care Medicine, Het Van Weel-Bethesda Ziekenhuis, Dirksland, The Netherlands; 65 Department of Intensive Care, Tergooi Hospital, Hilversum, The Netherlands

**Keywords:** Extubation, Prediction, Risk factors, Extubation failure

## Abstract

**Introduction:**

Determining the optimal timing for extubation can be challenging in the intensive care. In this study, we aim to identify predictors for extubation failure in critically ill patients with COVID-19.

**Methods:**

We used highly granular data from 3464 adult critically ill COVID patients in the multicenter Dutch Data Warehouse, including demographics, clinical observations, medications, fluid balance, laboratory values, vital signs, and data from life support devices. All intubated patients with at least one extubation attempt were eligible for analysis. Transferred patients, patients admitted for less than 24 h, and patients still admitted at the time of data extraction were excluded. Potential predictors were selected by a team of intensive care physicians. The primary and secondary outcomes were extubation without reintubation or death within the next 7 days and within 48 h, respectively. We trained and validated multiple machine learning algorithms using fivefold nested cross-validation. Predictor importance was estimated using Shapley additive explanations, while cutoff values for the relative probability of failed extubation were estimated through partial dependence plots.

**Results:**

A total of 883 patients were included in the model derivation. The reintubation rate was 13.4% within 48 h and 18.9% at day 7, with a mortality rate of 0.6% and 1.0% respectively. The grandient-boost model performed best (area under the curve of 0.70) and was used to calculate predictor importance. Ventilatory characteristics and settings were the most important predictors. More specifically, a controlled mode duration longer than 4 days, a last fraction of inspired oxygen higher than 35%, a mean tidal volume per kg ideal body weight above 8 ml/kg in the day before extubation, and a shorter duration in assisted mode (< 2 days) compared to their median values. Additionally, a higher C-reactive protein and leukocyte count, a lower thrombocyte count, a lower Glasgow coma scale and a lower body mass index compared to their medians were associated with extubation failure.

**Conclusion:**

The most important predictors for extubation failure in critically ill COVID-19 patients include ventilatory settings, inflammatory parameters, neurological status, and body mass index. These predictors should therefore be routinely captured in electronic health records.

**Supplementary Information:**

The online version contains supplementary material available at 10.1186/s13054-021-03864-3.

## Introduction

The decision to extubate a COVID-19 patient can be challenging and a delicate trade-off between early and postponed extubation. In non-COVID patients, extubation failure occurs in 10–20% of intensive care cases and is associated with increased mortality [1]. While postponing extubation and waiting for further clinical improvement appears sensible, unnecessary extubation delays may lead to more ventilator-associated complications and inefficient use of scarce intensive care resources [2, 3].

An understanding of the risk factors for extubation failure will aid the clinician in determining the optimal time point for extubation. Previous studies in non-COVID-19 patients have investigated numerous factors related to extubation outcome, including age, maximum inspiratory pressure, and the rapid shallow breathing index [4]. However, given the complex interplay of many patient and treatment related characteristics in extubation success, a single parameter rarely provides sufficient accuracy to guide decision making [5]. Moreover, it remains largely unclear whether these parameters are similar for COVID-19 patients [6].

The collection of large intensive care datasets that span the entire intensive care admission paves the way for machine learning models to capture this complex interplay of predictors by using machine learning models. Previous non-COVID-19 machine learning work has aimed to predict simple and difficult weaning [7] and extubation failure [8–15]. However, data was frequently from over a decade ago, mechanical ventilator data was usually lacking, and no data was included from COVID-19 patients. Taken together, we identify an opportunity for machine learning models to predict unsuccessful extubation in critically ill COVID-19 patients.

We created the Dutch Data Warehouse (DDW), a multicenter database with critically ill COVID-19 patients [16]. All structured electronic health record (EHR) data for these patients have been combined and cleaned for research purposes. These data therefore represent the structured EHR data readily available to the intensivist at the bedside. In this study, we aim to identify and validate the most important predictors for extubation failure in COVID-19 patients.

## Methods

This study follows the transparent reporting of a multivariable prediction model for individual prognosis or diagnosis (TRIPOD) guidelines [17].

### Data source

All data came from the DDW, a large, multicenter, full-admission, electronic health record data warehouse with data from critically ill COVID-19 patients in the Netherlands [16]. The data warehouse currently contains 3464 patients admitted between the beginning of the crisis in March 2020 and March 2021. Data spans both the first and second wave of ICU admissions from 25 hospitals in the Netherlands. The institutional review board of Amsterdam University Medical Center location VUmc waived the need for informed consent from individual patients and approved of an opt out procedure.

### Patients

All critically ill patients extubated after more than 24 h of invasive mechanical ventilation were eligible for inclusion. Transferred patients were included if the transfer destination data were available. We excluded patients transferred before extubation or within 1 day after extubation in case the transfer destination data were not available. Patients transferred more than 24 h after extubation were assumed to be fit for transport and classified as successful extubations. Patients still admitted at the time of data collection were excluded.

### Outcomes

The primary outcome was unsuccessful separation from invasive mechanical ventilation defined according to the WIND criteria [18], which mandate an extubation without reintubation or death within the next 7 days, or discharge from the ICU without invasive mechanical ventilation within 7 days [18]. The use of non-invasive ventilation is disregarded in this definition. As secondary outcomes, we applied the same criteria to a 48 h’ time window after extubation. The definition of extubation in EHR data has been published previously and reasonably excludes palliative care patients [16]. We did not distinguish between accidental and elective extubations as the reason for extubation is not routinely recorded.

### Predictors and scoping literature search

Potential predictors for modeling were selected by a team of intensivists. Notably, the list included medication and fluid balance. To facilitate the selection process, machine learning studies that predict extubation failure were identified in the literature. Each of the identified articles was scanned full-text and included predictors were extracted. The total list of studies can be found in Additional file 1: Table S1. In addition, to account for the wide variety of ventilator settings in the DDW, the parameters from the landmark paper by Amato et al. on the association between ventilator parameters and outcome were included in the selection [19]. The mean or last value from the last 24 h before extubation as specified by the team of intensivists were included to facilitate interpretation of the model. The total dose in the last 24 h was included for the medications. For any predictor pair with an interpredictor correlation higher than 0.5, the most clinically insightful predictor was selected. The full list of predictors can be found in Table 1.

**Table 1:** Included parameters

Predictor	Aggregation
Age	
Apache-II score	Mean last 24 h
Body mass index (BMI)	
C-reactive protein	Mean last 24 h
Creatinine	Mean last 24 h
FiO_2_	Mean last 24 h, Last value
Fluid balance	Sum
Glasgow coma scale	Mean last 24 h
Glucose	Mean last 24 h
Cardiac comorbidity	
Diabetes comorbidity	
Respiratory comorbidity	
Renal comorbidity	
Heart rate	Mean last 24 h
Hematocrit	Mean last 24 h
Gender	
Duration of mechanical ventilation	
Leukocyte count	Mean last 24 h
Benzodiazepine dose	Given in last 24 h
Clonidine dose	Given in last 24 h, Total dose last 24 h
Dexmedetomidine dose	Given in last 24 h, Total dose last 24 h
Fentanyl dose	Given in last 24 h, Total dose last 24 h
Haloperidol dose	Given in last 24 h, Total dose last 24 h
Midazolam dose	Given in last 24 h, Total dose last 24 h
Propofol dose	Given in last 24 h, Total dose last 24 h
Quetiapine dose	Given in last 24 h, Total dose last 24 h
p0.1	Mean last 24 h, Last value
P/F ratio	Mean last 24 h
PCO_2_ arterial	Last value
PEEP	Last value
pH	Last value
Hours since last proning session	
Pressure above PEEP	Mean last 24 h, Last value
Respiratory rate	Mean last 24 h
RASS score	Mean last 24 h, Last value
Thrombocyte count	Mean last 24 h
Tidal volume per kg ideal body weight	Mean last 24 h, Last value
Duration of controlled mode	
Hours since last controlled mode	
Ventilatory ratio	Mean last 24 h

Overview of included parameters and their aggregation

FiO_2_: Fraction of inspired oxygen, PEEP: positive end expiratory pressure, P/F ratio: PaO_2_/FiO_2_ ratio, PCO_2_: partial pressure of carbon dioxide, RASS score: Richmond Agitation and Sedation Scale

### Modeling

Across all 25 hospitals, a nested cross validation was performed to assess model performance. First, the data was split into five equally large sets called outer folds. These outer folds were then each split into a train and test set. Each of the train sets was again divided into five subsets called the inner folds. A model was trained on these 5 inner folds with a randomized hyperparameter search. Model performance after training on these inner folds was then tested on the corresponding outer fold test set. Importantly, observations belonging to the same patient were always kept in the same split to prevent leakage of information. The overall model performance was the average of all outer fold test set performances.

We trained a logistic regression model, decision trees, and an XGBoost algorithm. These models were selected for their ease of determining predictor importance. Model performance was gauged with the area under the receiver operating characteristic (AUROC), Brier score, average precision, and calibration loss. Data imputation, standardization and automated feature selection were carried out on each outer fold separately. Missing values were imputed with the median and predictors were standardized to have a mean of 0 and a standard deviation of 1. Lasso regression was performed for automatic feature selection, and the L1 regularization term was optimized together with the other hyperparameters [20].

Predictor importance was estimated with the Shapley additive explanation (SHAP) framework. SHAP values represent a predictor’s marginal contribution to the overall prediction [21] and are state of the art in machine learning explainability. Moreover, Partial Dependence Plots (PDPs) were created to visualize the average change in probability of successful extubation for all values of a predictor while keeping all other predictors constant [22]. Standard deviations represent the distribution of the data. All analyses were carried out in Python 3.8 (Python software foundation).

## Results

### Population and outcome

A total of 2.421 patients were mechanically ventilated during their ICU stay. In case of a patient transfer, data from the transferring and receiving hospital were merged when available. We excluded 517 transfers for which outcome or admission data were lacking, 123 patients that were still intubated when data were extracted, and 139 patients that were intubated less than 24 h. 568 patients died on the mechanical ventilator before their first extubation attempt and 191 patients received a tracheostomy. As a result, a total of 883 patients were included in the modeling. The reintubation rate in this COVID-19 population was 18.9% within 7 days and 13.4% within 48 h. The mortality rate was 1.0% within 7 days and 0.6% in the first 48 h after extubation. Patient characteristics are outlined in Table 2.

**Table 2:** Patient characteristics

	Total patients (*N* = 883)	Successful extubation (*N* = 707)	Unsuccessful extubation (*N* = 176)
Male	71.5% (*N* = 883)	70.4% (*N* = 707)	75.6% (*N* = 176)
Age, years	63 (55–70, *N* = 883)	63 (55–69, *N* = 707)	65 (57–72, *N* = 176)
*Age, years*			
< 60	360 (40.8%)	301 (42.6%)	59 (33.5%)
60–70	314 (35.6%)	249 (35.2%)	65 (36.9%)
70–80	199 (22.5%)	150 (21.2%)	49 (27.8%)
> 80	10 (1.1%)	7 (1.0%)	3 (1.7%)
Body mass index, kg/m^2^	27.9 (25.1–31.6, *N* = 809)	28.4 (25.4–32.0, *N* = 642)	26.8 (24.2–30.1, *N* = 167)
*Body mass index, kg/m*^*2*^			
< 25	200 (24.7%)	145 (22.6%)	55 (32.9%)
25–30	336 (41.5%)	267 (41.6%)	69 (41.3%)
30–35	174 (21.5%)	145 (22.6%)	29 (17.4%)
> 35	98 (12.1%)	84 (13.1%)	14 (8.4%)
*Lab values (last 24 h before extubation)*			
C-reactive protein, mg/L	57 (23–114, *N* = 731)	53 (21–108, *N* = 583)	72 (33–146, *N* = 148)
Creatinine, micromol/L	66 (52–96, *N* = 820)	65 (52–90, *N* = 657)	66 (53–108, *N* = 163)
Leukocyte count, 10^9^/L	11.3 (8.8–14.2, *N* = 817)	11.2 (8.8–13.9, *N* = 653)	11.7 (8.9–15.5, *N* = 164)
Thrombocyte count, 10^9^/L	350 (269–457, *N* = 824)	356 (269–462, *N* = 664)	340 (259–419, *N* = 160)
*Respiratory characteristics (last measured)*			
Time since last controlled mode, hours	74 (35–130, *N* = 773)	77 (37–137, *N* = 603)	64 (27–115, *N* = 170)
FiO_2_, %	36 (30–41, *N* = 866)	35 (30–41, *N* = 691)	40 (33–45, *N* = 175)
Pressure above PEEP, cmH_2_O	6 (5–9, *N* = 773)	6 (5–10, *N* = 617)	7 (5–8, *N* = 156)
PEEP, cmH_2_O	8 (5–8, *N* = 867)	8 (6–9, *N* = 693)	7 (5–8, *N* = 174)
Respiratory rate, /min	22 (18–26, *N* = 883)	22 (18–26, *N* = 707)	23 (18–27, *N* = 176)
Tidal volume, ml/kg IBW	7.5 (6.4–9.0, *N* = 857)	7.5 (6.3–8.9,* N* = 682)	7.6 (6.6–9.1,* N* = 175)
Ventilatory ratio	1.8 (1.4–2.3,* N* = 722)	1.8 (1.4–2.3,* N* = 577)	1.9 (1.5–2.4,* N* = 145)
P/F ratio	206 (168–258,* N* = 861)	209 (171–262,* N* = 690)	192 (163–242,* N* = 171)
pO_2_ arterial, mmHg	74 (67–84,* N* = 792)	75 (68–85,* N* = 634)	72 (65–83,* N* = 158)
pCO_2_ arterial, mmHg	41 (37–46,* N* = 732)	41 (37–46,* N* = 579)	40 (36–46,* N* = 153)
Bicarbonate arterial, mmol/L	29 (26–31,* N* = 861)	29 (26–32,* N* = 688)	28 (25–31,* N* = 173)
Airway occlusion pressure (P0.1), cmH_2_O	2.1 (1.3–3.8,* N* = 450)	2.1 (1.2–3.7,* N* = 356)	2.3 (1.4–4.0,* N* = 94)

Overview of patient characteristics, lab characteristics and ventilatory characteristics before extubation. All values are medians with an interquartile range, unless otherwise indicated

FiO_2_: Fraction of inspired oxygen, PEEP: positive end expiratory pressure, P/F ratio: PaO_2_/FiO_2_ ratio, IBW: ideal body weight, PO_2_: partial pressure of oxygen, PCO_2_: partial pressure of carbon dioxide

### Modeling

Model performance for the primary outcome is shown in Additional file 1: Table S2 for each of the models. The XGBoost algorithm yielded the highest performance with an AUROC of 0.70, outperforming logistic regression (AUROC 0.67) and a decision tree (AUROC 0.59). Model performance for the prediction of unsuccessful extubation 48 h after extubation is presented in Additional file 1: Table S2. All algorithms, XGBoost (AUROC 0.67), logistic regression (0.66), and a decision tree (AUROC 0.54), performed worse compared to the primary outcome.

### Predictor importance

**Fig. 1:**
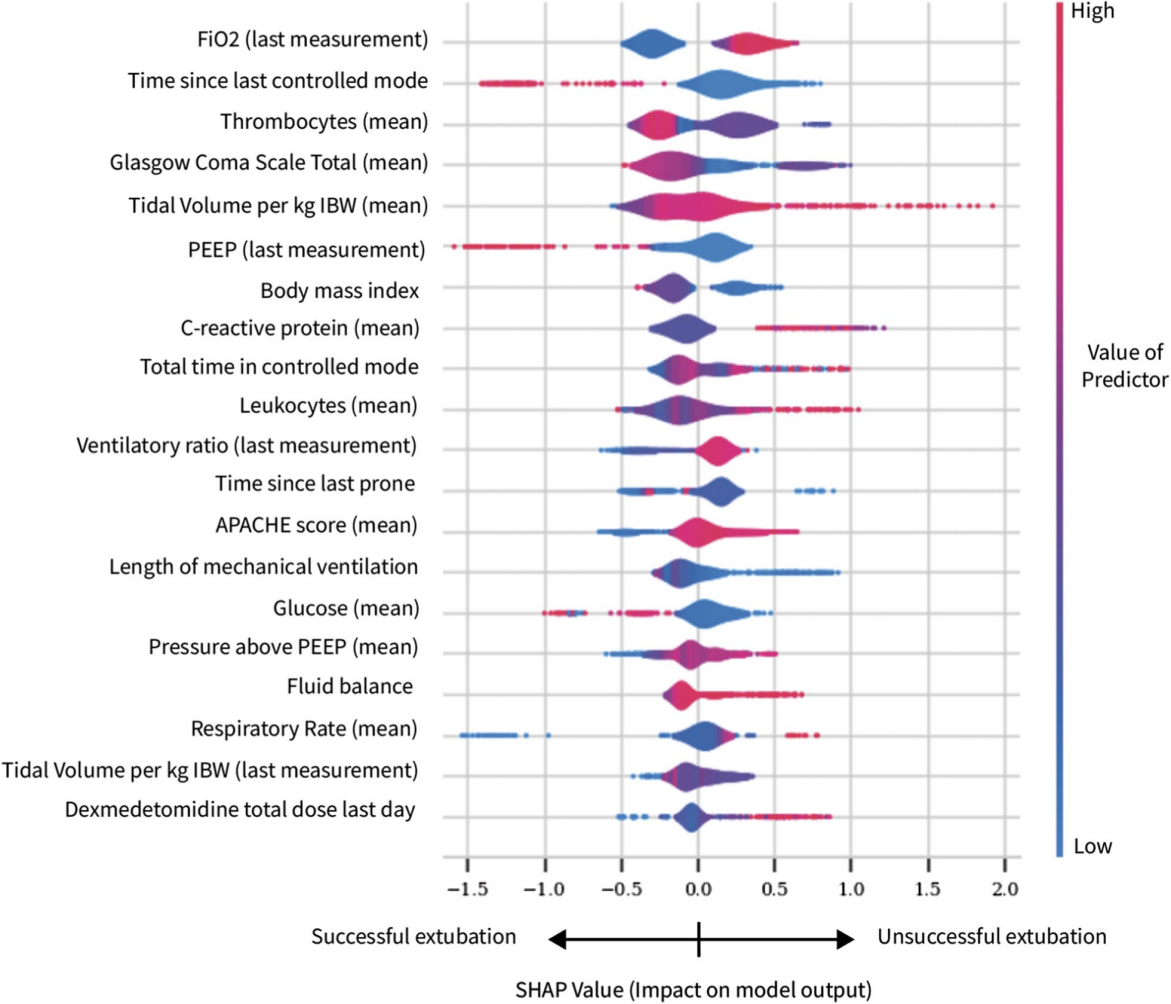
SHAP values for most important predictors of extubation failure. Overview of SHAP values for the top 20 predictors of successful extubation (negative SHAP values) or unsuccessful extubation (positive SHAP values). Features are ordered according to importance. FiO2: fraction of inspired oxygen, IBW: ideal body weight, PEEP: positive end expiratory pressure, P/F ratio: PaO2/FiO2 ratio

Predictor importance was calculated with the XGBoost model since it yielded the highest performance. The SHAP values for the highest predictors are shown in Fig. 1. The most important predictive feature of extubation failure was the last FiO_2_ value before extubation. The majority of important predictors can be grouped into ventilatory characteristics, inflammation markers, neurological status and body mass index.

### Ventilatory characteristics

Ventilatory characteristics are shown in Table 2. A short time-period between the last controlled mode and extubation, and a longer duration in controlled mode throughout the course of mechanical ventilation were associated with unsuccessful extubation. The PD-plots depict the difference in predicted probability of extubation failure compared to the median value for all of the observed values. The PD-plot shows a time since the last controlled mode shorter than 2 days and a controlled mode duration longer than 4 days are associated with increased chances of unsuccessful extubation compared to the median value.

**Fig. 2:**
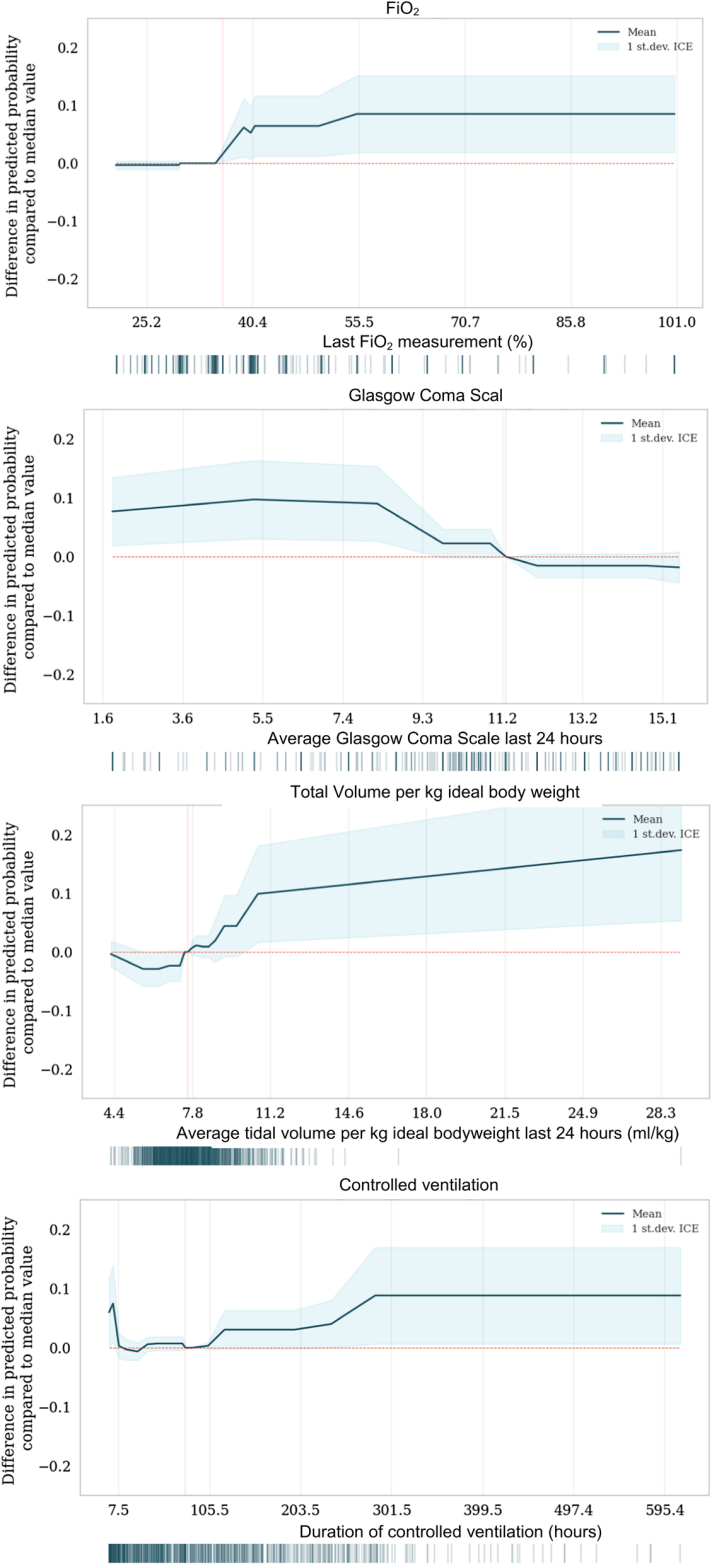
Partial dependence plots. PD-plot for the last FiO2 recording, mean glasgow coma score and tidal volume per kg ideal body weight in the last 24 h, and duration of the controlled mode

For the ventilator settings, a higher fraction of inspired oxygen and a higher average tidal volume in the last 24 h are predictive of extubation failure. The PD-plot in Fig. 2 shows that an FiO_2_ above 35% or a tidal volume per kg ideal body weight above 8 ml/kg compared to their median values increases the probability of unsuccessful extubation. The median PEEP was 8 cmH_2_O (IQR 5–8 cmH_2_O) before extubation, with a median pressure support of 6 cmH_2_O (IQR 5–9 cmH_2_O). No patients received PEEP levels below 5 cmH_2_O, while pressure above PEEP was below 5 cmH_2_O in 7.3% of patients.

### Inflammation markers, neurological scores and body mass index

Both a higher CRP, an elevated leukocyte count and higher thrombocyte count in the 24 h preceding extubation are predictors of an unsuccessful extubation attempt, while temperature was not in the top predicting features. For neurological scores, on the other hand, low EMV scores predict unsuccessful extubation. Lastly, BMI showed an inverse relationship with extubation failure; patients with a higher BMI had a lower probability of extubation failure. An increase in the chances of unsuccessful extubation is observed below 28 kg/m^2^ compared to the median in the PD-plot (shown in Additional file 1: Fig. S1).

## Discussion

To the best of our knowledge, this is the first study that identifies predictors for extubation failure in critically ill COVID-19 patients from a large and multicenter cohort that contains a wide variety of routinely collected clinical predictors. The most important predictors of extubation failure are ventilatory characteristics, inflammatory parameters, GCS score, and body mass index. These risk factors may aid intensive care professionals in selecting the optimal time point for extubation.

This study is unique as it provides predictive modeling of extubation failure across twenty-five hospitals. All previous machine learning studies in non-COVID patients for predicting extubation failure have been single center [7–15]. Model performance was higher in these studies, presumably due to overfitting resulting from the sole use of local data. Algorithms may be biased towards local extubation practices and extubation readiness assessments, making these models less generalizable to other clinical settings.

In our study, ventilatory characteristics, including ventilator settings, are the most important risk factors for extubation failure. These factors are systematically and frequently recorded by the ventilators, and are potentially modifiable. Two of the most important predictors associated with higher chances of failed extubation are the duration of the controlled and assisted ventilation modes prior to extubation. A longer time in a controlled mode was a stronger predictor than the total duration of mechanical ventilation. Moreover, a longer time in assisted mode was associated with improved chances of successful extubation. A possible explanation may be the reduced activity and consequent atrophy of the diaphragm or other skeletal muscles in controlled modes [23, 24]. Of note, none of the previous machine learning studies included the duration of controlled ventilation as a predictor. Our results show that the duration of ventilation modes should be recorded and taken into account when assessing extubation readiness.

For the ventilator settings, a higher FiO_2_ before extubation was associated with an increased risk of extubation failure. A higher FiO_2_ may indicate incomplete resolution of pulmonary pathology. Higher PEEP levels, on the other hand, were associated with better extubation success. The interquartile ranges of PEEP are low, however, indicating low PEEP is common practice before an extubation attempt. In addition, we observed that higher mean tidal volumes corrected for the ideal body weight in the last day before extubation were an important predictor of extubation failure. Patients with high average tidal volumes may suffer from more lung injury that may increase the risk of unsuccessful extubation [25]. While most of the ventilator settings are readily available, relevant respiratory system maneuvers such as spontaneous breathing trials, tracheobronchial suctioning and maximum inspiratory pressure that would ideally be included, were inconsistently recorded in the EHR systems and therefore not included in modelling. To evaluate their predicting importance in extubation failure, data of these maneuvers need to be incorporated systematically in the EHR.

Other important predictors included signals of ongoing or developing inflammation, poorer neurological status, and body mass index. Inflammation parameters are routinely determined in most intensive care units when extubation decisions are made. Conversely, neurological scores can be ambivalently scored in the intensive care unit. The Glasgow Coma Scale was originally designed for brain damage patients [26], but is used for the general intensive care patient. Unequivocal interpretation of sedated states, however, may hamper the use of this scale in the context of extubation readiness. Based on these results, we would recommend systematically recording and evaluating the predictive value of other scores like the Richmond agitation sedation scales.

Lastly, body mass index upon admission had an inverse relationship with extubation failure. Apart from one small study that found an association between BMI and post extubation stridor [27], no other studies were identified that found BMI to be an important predictor. As in any predictive study, the effect of BMI may be explained by an unmeasured predictor or a selection bias. That means, a low-BMI patient would have to be sicker to be admitted to the ICU. A negligible correlation was found between BMI and SOFA score, however, as an indicator of illness severity. Previous studies have also shown that BMI is uncorrelated with immunological responses or adverse outcomes [28]. Overall, once in the ICU, BMI is not related to higher chances of unsuccessful extubation and may not be a valid reason to postpone extubation.

Our study has several limitations. We aim to apply a holistic set of predictors across centers to assess extubation readiness. In routine practice, however, individualized treatment and diagnostic decisions result in variation of available parameters [29], and predictors may be unavailable in the 24 h prior to extubation. For example, it is not possible to conclude that cardiac markers like NT-pro-BNP or troponin do not aid in the prediction of extubation failure, because these markers were not routinely determined. Along the same line, we had to merge groups of medications, because individual drugs may not be administered frequently enough to be useful in the modeling. To truly exploit the predictive power of machine learning models, we should strive to systematically record the predictors of interest and determine which algorithms work in what clinical circumstances [30].

A further limitation is the missing outcome data because of patient transfers to centers not included in this project. The potential bias is considered small, as we connected all patients’ stays whenever available and transferred patients had similar baseline characteristics as the study population as a whole [31]. Lastly, the relationships identified in this study are associations and do not equal causation. As with any clinical observational dataset, we cannot observe counterfactual states; once a patient is extubated we irretrievably lose the outcome in case the patient would have been kept on mechanical ventilation. While many of the ventilatory settings are predictive of extubation failure, we would ultimately be interested in the effects of continuing mechanical ventilation for another day on extubation success. We believe that these results will provide a crucial step for other study designs to investigate the causal relation between modifiable predictors and successful extubation.

## Conclusion

This is the first study to identify risk factors of extubation failure in a large multi-center cohort of critically ill COVID-19 patients. The large number of hospitals included limits the risk of overfitting due to specific local practices. From a large set of clinically important predictors, ventilatory characteristics, inflammatory markers, neurological status and BMI were most important predictors for failed extubation. These predictors should be taken into account to determine extubation readiness.

## Supplementary Information

**Additional file 1**. **Table S1** Overview of machine learning studies that investigate extubation readiness. **Table S2** Model performance. **Figure S1** PD-plot for BMI

## Data Availability

The Dutch Data Warehouse is available for global collaboration, within the restrictions imposed by privacy laws and ethics. Information on how to access the data warehouse may be found at www.amsterdammedicaldatascience.nl.

## Abbreviations

APACHE II: Acute physiology and chronic health evaluation iI
AUROC: Area under the receiver operating characteristics curve
BMI: Body mass index
CRP: C-reactive protein
DDW: Dutch data warehouse
EHR: Electronic health record
EMV score: Eye motor verbal score
FiO_2_: Fraction of inspired oxygen
GCS: Glasgow coma scale
IQR: Interquartile range
PDP: Partial dependence plot
PEEP: Positive end expiratory pressure
RASS: Richmond agitation and sedation scales
SHAP: Shapley additive explanation
SOFA: Sequential organ failure assessment

## References

[1] Thille AW, Richard J-CM, Brochard L (2013). The decision to extubate in the intensive care unit. Am J Respir Crit Care Med.

[2] Slutsky AS, Ranieri VM (2013). Ventilator-induced lung injury. N Engl J Med.

[3] Ventilator-Associated Events: Prevalence, Outcome, and Relat ionship With Ventilator-Associated Pneumonia. Critical Care Medicine [Internet]. [cited 2021 Jun 17]. Available from: https://journals.lww.com/ccmjournal/Abstract/2015/09000/Ventilator_Associated_Events__Prevalence,_Outcome,.3.aspx

[4] Baptistella AR, Sarmento FJ, da Silva KR, Baptistella SF, Taglietti M, Zuquello RÁ (2018). Predictive factors of weaning from mechanical ventilation and extubation outcome: a systematic review. J Crit Care.

[5] Heunks LM, van der Hoeven JG (2010). Clinical review: The ABC of weaning failure—a structured approach. Crit Care.

[6] Ionescu F, Zimmer MS, Petrescu I, Castillo E, Bozyk P, Abbas A (2021). Extubation failure in critically ill COVID-19 patients: risk factors and impact on in-hospital mortality. J Intensive Care Med.

[7] Hsieh MH, Hsieh MJ, Cheng A-C, Chen C-M, Hsieh C-C, Chao C-M (2019). Predicting weaning difficulty for planned extubation patients with an artificial neural network. Medicine (Baltimore).

[8] Fabregat A, Magret M, Ferré JA, Vernet A, Guasch N, Rodríguez A (2021). A machine learning decision-making tool for extubation in Intensive care unit patients. Comput Methods Programs Biomed.

[9] Hsieh M-H, Hsieh M-J, Chen C-M, Hsieh C-C, Chao C-M, Lai C-C (2018). An artificial neural network model for predicting successful extubation in intensive care units. J Clin Med.

[10] Tsai T-L, Huang M-H, Lee C-Y, Lai W-W (2019). Data science for extubation prediction and value of information in surgical intensive care unit. J Clin Med.

[11] Lin M-Y, Li C-C, Lin P-H, Wang J-L, Chan M-C, Wu C-L (2021). Explainable machine learning to predict successful weaning among patients requiring prolonged mechanical ventilation: a retrospective cohort study in central Taiwan. Front Med.

[12] Jia Y, Kaul C, Lawton T, Murray-Smith R, Habli I (2021). Prediction of weaning from mechanical ventilation using convolutional neural networks. Artif Intell Med.

[13] Zhao Q-Y, Wang H, Luo J-C, Luo M-H, Liu L-P, Yu S-J (2021). Development and validation of a machine-learning model for prediction of extubation failure in intensive care units. Front Med.

[14] Kuo H-J, Chiu H-W, Lee C-N, Chen T-T, Chang C-C, Bien M-Y (2015). Improvement in the prediction of ventilator weaning outcomes by an artificial neural network in a medical ICU. Respir Care.

[15] Otaguro T, Tanaka H, Igarashi Y, Tagami T, Masuno T, Yokobori S (2021). Machine learning for the prediction of successful extubation among patients with mechanical ventilation in the intensive care unit: a retrospective observational study. J Nippon Med Sch Nippon Ika Daigaku Zasshi.

[16] Fleuren LM, Dam TA, Tonutti M, de Bruin DP, Lalisang RCA, Gommers D (2021). The Dutch data warehouse, a multicenter and full-admission electronic health records database for critically ill COVID-19 patients. Crit Care.

[17] Collins GS, Reitsma JB, Altman DG, Moons KGM (2015). Transparent reporting of a multivariable prediction model for individual prognosis or diagnosis (TRIPOD): the TRIPOD statement. BMJ.

[18] Béduneau G, Pham T, Schortgen F, Piquilloud L, Zogheib E, Jonas M (2016). Epidemiology of weaning outcome according to a new definition. The WIND study. Am J Respir Crit Care Med.

[19] Amato MBP, Meade MO, Slutsky AS, Brochard L, Costa ELV, Schoenfeld DA (2015). Driving pressure and survival in the acute respiratory distress syndrome. N Engl J Med.

[20] Tibshirani R (1996). Regression Shrinkage and Selection via the Lasso. J R Stat Soc Ser B Methodol.

[21] Chen H, Janizek JD, Lundberg S, Lee S-I. True to the Model or True to the Data? ArXiv200616234 Cs Stat [Internet]. 2020 [cited 2021 Jan 28]; Available from: http://arxiv.org/abs/2006.16234

[22] Friedman JH (2001). Greedy function approximation: a gradient boosting machine. Ann Stat.

[23] Rapid Disuse Atrophy of Diaphragm Fibers in Mechanically Ventilated Humans | NEJM [Internet]. [cited 2021 Sep 21]. Available from: 10.1056/nejmoa070447 10.1056/NEJMoa070447 18367735

[24] Mechanical Ventilation–induced Diaphragm Atrophy Strongly Impacts Clinical Outcomes | American Journal of Respiratory and Critical Care Medicine [Internet]. [cited 2021 Sep 21]. Available from: 10.1164/rccm.201703-0536OC 10.1164/rccm.201703-0536OC 28930478

[25] Yoshida T, Amato MBP, Kavanagh BP, Fujino Y (2019). Impact of spontaneous breathing during mechanical ventilation in acute respiratory distress syndrome. Curr Opin Crit Care.

[26] Teasdale G, Jennett B (1974). Assessment of coma and impaired consciousness: a practical scale. The Lancet.

[27] Erginel S, Ucgun I, Yildirim H, Metintas M, Parspour S (2005). High body mass index and long duration of intubation increase post-extubation stridor in patients with mechanical ventilation. Tohoku J Exp Med.

[28] Kooistra EJ, de Nooijer AH, Claassen WJ, Grondman I, Janssen NAF, Netea MG (2021). A higher BMI is not associated with a different immune response and disease course in critically ill COVID-19 patients. Int J Obes.

[29] Qian Z, Alaa AM, van der Schaar M, Ercole A (2020). Between-centre differences for COVID-19 ICU mortality from early data in England. Intensive Care Med.

[30] Eaneff S, Obermeyer Z, Butte AJ (2020). The case for algorithmic stewardship for artificial intelligence and machine learning technologies. JAMA.

[31] Fleuren LM, de Bruin DP, Tonutti M, Lalisang RCA, Elbers PWG, Gommers D (2021). Large-scale ICU data sharing for global collaboration: the first 1633 critically ill COVID-19 patients in the Dutch Data Warehouse. Intensive Care Med.

