# Supplementary material for: Predictors for extubation failure in COVID-19 patients using a machine learning approach
Source: Crit Care. 2021 Dec 27;25:448. doi: 10.1186/s13054-021-03864-3 (PMC8711075; doi:10.1186/s13054-021-03864-3)
Supplement: Supplementary file 1 — Additional file 1. Table S1 Overview of machine learning studies that investigate extubation readiness. Table S2 Model performance. Figure S1 PD-plot for BMI [file 13054_2021_3864_MOESM1_ESM.docx]

# Additional File

**Table 1. Overview of machine learning studies that investigate extubation readiness**

| **Authors** | **Year** | **Model** | **Outcome** | **Patients** | **Predictors** |
| --- | --- | --- | --- | --- | --- |
| Kuo et al. | 2015 | ANN | Reintubation <48 hours | 121 | 8 |
| Hsieh et al. | 2018 | ANN | Reintubation <72 hours | 3602 | 37 |
| Hsieh et al. | 2019 | ANN | Simple, difficult, prolonged weaning | 3602 | 57 |
| Fabregat et al. | 2020 | LDA, GBM, SVM | Reintubation <48 hours | 1570 | 19 |
| Lin et al. | 2021 | XGBoost, RF, LR | Reintubation <120 hours | 963 | 300 |
| Otaguro et al. | 2021 | RF, XGBoost, LightGBM | Reintubation <72 hours | 117 | 58 |
| Jia et al. | 2021 | CNN | Reintubation <48 hours | 2299 | 24 |
| Zhao et al. | 2021 | CatBoost | Reintubation <48 hours | 16189 | 78 |

*ANN: Artificial neural network, LDA: Logistic discriminant analysis, GBM: Gradient boosting method, SVM: Support vector machines, RF: Random Forest, CNN: Convolutional neural network, LR: Logistic regression.*

*Search strategy:*

| Machine learning | "Artificial Intelligence"[Mesh] OR machine learning[ti] OR prediction algorithm*[ti] OR prediction model*[ti] OR neural network*[ti] OR deep learning[ti] OR artificial intelligence[ti] OR AI[ti] OR decision tree*[ti] OR computational intelligence[ti] OR machine intelligence[ti] OR big data[ti] OR bayesian[ti] OR naïve bayes[ti] OR k-nearest neighbour[ti] OR random forest[ti] OR support vector machine[ti] OR SVM[ti] OR Xgboost[ti] OR adaboost[ti] OR gradient boosting machine*[ti] OR regression tree*[ti] OR least squares[ti] OR stepwise regression[ti] |
| --- | --- |
| Extubation | "Airway Extubation"[Mesh] OR "Ventilator Weaning"[Mesh] OR extubation[ti] OR weaning[ti] |
| Adults | NOT (("Adolescent"[Mesh] OR "Child"[Mesh] OR "Infant"[Mesh] OR adolescen*[tiab] OR child*[tiab] OR schoolchild*[tiab] OR infant*[tiab] OR girl*[tiab] OR boy*[tiab] OR teen[tiab] OR teens[tiab] OR teenager*[tiab] OR youth*[tiab] OR pediatr*[tiab] OR paediatr*[tiab] OR puber*[tiab]) NOT ("Adult"[Mesh] OR adult*[tiab] OR man[tiab] OR men[tiab] OR woman[tiab] OR women[tiab])) NOT (animals[mh] NOT humans[mh]) |

*The search was performed in Pubmed and is up to date until 16-09-2021*

**Table 2. Model performance**

| **Model** | **ROC-AUC** | **Brier score** |
| --- | --- | --- |
| Unsuccesful extubation - 7 days |  |  |
| - Decision Tree | 0.59 ± 0.043 | 0.17 ± 0.007 |
| - Logistic Regression | 0.67 ± 0.032 | 0.16 ± 0.004 |
| - XGBoost | 0.70 ± 0.028 | 0.15 ± 0.007 |
| Unsuccesful extubation - 48 hours |  |  |
| - Decision Tree | 0.54 ± 0.059 | 0.13 ± 0.004 |
| - Logistic Regression | 0.66 ± 0.038 | 0.12 ± 0.007 |
| - XGBoost | 0.67 ± 0.006 | 0.12 ± 0.003 |

*Model performance for each of the models, for unsuccessful extubation within 7 days and 48 hours*

**Figure 1. PD-plot for BMI**

Body mass index

**
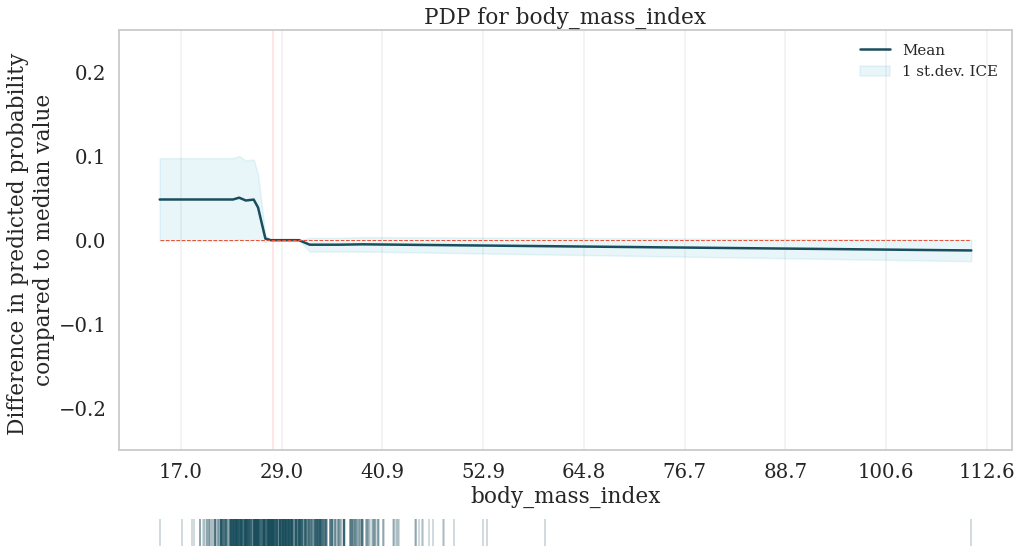
**

Body mass index (kg/m^2^)
